# Artificial intelligence applied to local production of diagnostic tests

**DOI:** 10.2471/BLT.25.293235

**Published:** 2025-10-23

**Authors:** Jicui Dong, David Woo, Vasileia Kostaridou, Yan Wang, Virginia Maria Ferreira Resende

**Affiliations:** a Health Products Policy and Standards Department, World Health Organization, Avenue Appia 20, 1211 Geneva 27, Switzerland.

In vitro diagnostic medical devices are a critical component of health systems. Applications extend beyond disease diagnosis and include screening, classification, prognosis and monitoring, therapy selection, patient monitoring and determination of pathological and physiological states. In vitro diagnostics enhance patient outcomes and reduce health-care costs because they enable rapid, cost-effective and point-of-care diagnoses. Therefore, during a pandemic, in vitro diagnostics are key in rapid identification and tracking of infectious agents, ensuring timely treatments and outbreak control.

The production value chain and lifecycle of in vitro diagnostics involves interconnected stages, from research and development, manufacturing and supply chain management to marketing and sales, post-market surveillance and regulatory compliance. Low- and middle-income countries face many challenges along the value chain that can affect their capacity for local production of quality-assured in vitro diagnostics. For example, many of these countries face a shortage of experts and a skilled manufacturing workforce, fragmented markets and weak logistics infrastructure and data collection systems.^1^ Regulatory fragmentation, combined with low maturity levels of regulatory systems in these countries, contribute to slow approval processes and inconsistent enforcement, hindering the timely entry of quality products into the market.^1^ In vitro diagnostic manufacturers in low- and middle-income countries also face challenges to conduct research and development and achieve consistent quality and production efficiencies and scale-up.

This article highlights the evolving trends and untapped opportunities of artificial intelligence (AI) in enhancing local in vitro diagnostic production, paving the way for future adoption by manufacturers. Concrete examples of adopting AI in local in vitro diagnostic production in the public domain are currently limited. However, just as it is being adopted in pharmaceuticals and medical devices,^2^^,^^3^ AI holds considerable potential for manufacturers to overcome challenges across the in vitro diagnostic production value chain and lifecycle with diverse tools (Fig. 1). Machine learning enables systems to improve autonomously without explicit programming. Deep learning, a subset of machine learning, uses multilayered neural networks for data analysis. Generative AI creates new content, such as text, images, audio or video, rather than simply analysing existing data. Large multimodal models, a subtype of generative AI,^4^ are trained on diverse data sets to process multiple input types and generate varied outputs, making them suitable for a broad range of tasks and applications. Other emerging AI technologies include digital twins, smart computer-aided design, AI-driven robotics, automated quality inspections and AI-assisted reporting. AI-powered platforms facilitate data collection, storage, analysis and dissemination, therefore improving decision-making and operational efficiency. AI-driven data analytics enable the examination of extensive data sets to optimize manufacturing processes. Cloud computing offers scalable infrastructure for improved data storage, processing and collaboration, while smart device integration through connected technologies enables automation and real-time quality control.^5^ Additionally, blockchain technology provides secure and tamper-proof records, allowing manufacturers to track raw materials and finished products with transparency.

**Fig. 1:**
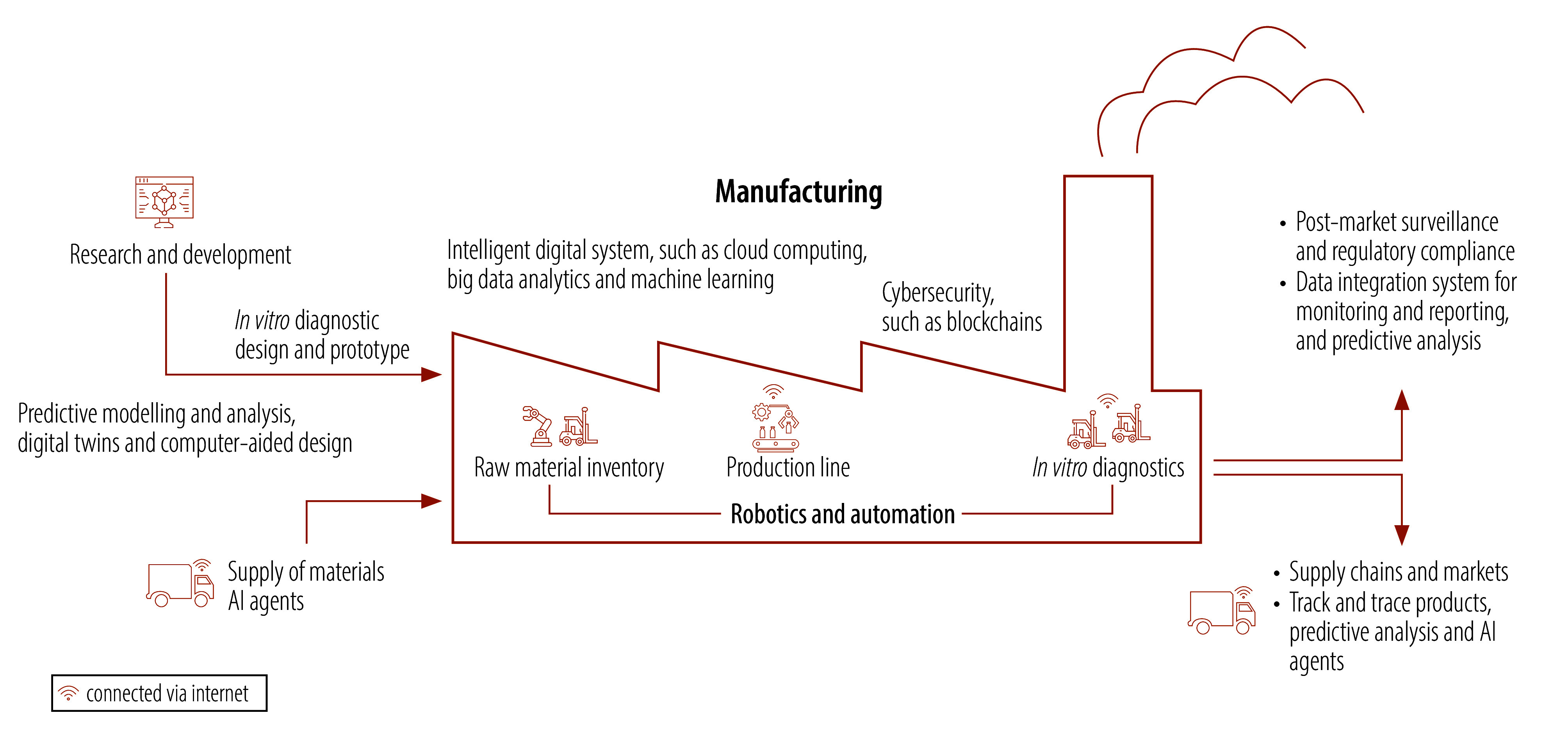
Outlook for integrating AI technologies into local in vitro diagnostic production across the value chain and lifecycle

Note: an AI agent is an autonomous entity (software or hardware) that can perceive its environment, process data, make decisions and then take action to achieve specific goals.

The projected integration of AI technologies in local in vitro diagnostic production has the potential to drive transformation and innovation across the value chain and product lifecycle. Machine learning algorithms can identify biomarkers and disease patterns, accelerating innovation. AI-driven predictive modelling can analyse historical data to enhance research and development by predicting how new approaches will perform or simulate clinical trials and predict clinical outcomes. In in vitro diagnostic design and prototyping, digital twins and AI-assisted computer-aided design can optimize design variations,^6^ by creating detailed three-dimensional models and simulating their behaviour under different situations and conditions. This capacity allows for thorough testing, reduced errors and better validation of designs and prediction of potential failure scenarios before physical prototyping.

AI-driven automation and robotics in medical device manufacturing enhance precision, consistency and efficiency while reducing human errors. These technologies demonstrate considerable transferability to in vitro diagnostic production, where similar demands for process accuracy and optimization exist. For instance, AI-driven tools could adapt to changes during production and adjust the manufacturing parameters in real-time to maintain optimal conditions. Predictive maintenance may anticipate equipment failures, minimizing downtime, thereby reducing maintenance costs. AI-based quality control systems can use connected sensors and devices to continuously monitor critical parameters and manufacturing conditions, enabling real-time detection of defects. By integrating data systems, cloud computing and AI-driven analytics, large volumes of production data, from internet-connected sensors, production logs and quality control test results, can be centralized, stored and analysed. In turn, this process enables the identification of manufacturing and product quality trends, facilitates historical comparisons and generates regulatory reports.

Supply chain optimization is crucial for ensuring a stable flow of raw materials and finished products. In the current digital age, companies can access global supply chain networks online and track orders. AI tools, such as AI agents, can help to enhance search capabilities to find the right suppliers. AI-driven predictive analytics improve demand forecasting through utilization of historical sales data, seasonal patterns and market dynamics. AI algorithms can further streamline inventory management (for example, automated replenishment of raw materials), logistics and supply route optimization, thereby reducing costs in supply chain management.^7^ These approaches are readily transferable to in vitro diagnostic supply chains, where AI could optimize time-sensitive reagent deliveries and mitigate supply disruptions for critical diagnostic components.

AI also supports manufacturers in navigating complex and evolving regulatory landscapes. Regulatory compliance can be enhanced by automating documentation, analysing regulatory requirements, flagging inconsistencies, monitoring of regulatory changes and adapting to new guidelines in a timely manner. Integrating AI into clinical studies can facilitate recording, monitoring and analysis of clinical data, predict clinical outcomes and potential issues, and automate compliance reporting. In post-market surveillance, AI can uncover patterns of performance issues and long-term safety concerns, allowing manufacturers to optimize safety and performance throughout the product lifecycle.^8^

Prospective integration of AI tools into local in vitro diagnostic production, particularly in low- and middle-income countries, may encounter multiple implementation barriers. Manufacturers face limitations in technical capacity when integrating AI into existing manufacturing processes, including data integration, system interoperability and maintaining data quality. Moreover, adopting technologies such as AI, internet-connected devices and blockchain involves considerable financial investments in new machinery, structures and expertise. Regulatory approval for AI applications remains complex due to the lack of standardized frameworks, particularly for generative AI, which can create synthetic data that is nearly indistinguishable from real-world data.^9^ The dynamic nature of AI algorithms, especially those that evolve new data (adaptive AI), coupled with the lack of standards and a robust regulatory framework, pose challenges for regulatory bodies in conducting the conformity assessment and demonstrating transparency. In parallel, manufactures face challenges with generating high-quality clinical evidence, ensuring that AI systems consistently meet safety and effectiveness standards.

The envisioned adoption of AI in local in vitro diagnostic production also raises significant ethical concerns that must be carefully addressed to ensure responsible and equitable application. Ethical concerns regarding fairness, accountability and potential biases in AI decision-making also require attention. AI systems should be designed and used to promote equity, inclusivity and accountability while preserving human dignity, values and rights. The World Health Organization published two reports in 2021 and 2023^9^^,^^10^ outlining key principles for the ethical and responsible use of AI systems in health care. The importance of data privacy and protection is critical when adopting AI and digital technologies, particularly with the rise of internet-connected devices and AI applications that handle large amounts of confidential data such as records of clinical trial subjects and proprietary research. While blockchains strengthen security and accountability with their decentralized and immutable record-keeping features, it brings about new challenges in maintaining data governance and adhering to data privacy or protection laws.^11^ Lastly, human oversight has become a focal point in international AI governance debates, yet the feasibility of maintaining meaningful expert human oversight remains a critical challenge given the increasing complexity of AI architectures.^12^

Strengthening local production diversifies production capacity and capability, reduces supply chain disruptions and minimizes reliance on imports, ensuring timely and equitable access to essential medical products, particularly during health crises. Recognizing the importance of local production as a strategy to ensure availability, affordability and accessibility of essential medical products, the World Health Assembly adopted the Resolution WHA74.6, *Strengthening local production of medicines and other health technologies to improve access*, emphasizing the need to enhance production capabilities in low- and middle-income countries.^13^

While deployment and integration challenges exist, in vitro diagnostic manufacturers in low- and middle-income countries could leverage AI technology to potentially boost research and development as well as production capacity and efficiency. Doing so would also reduce costs, enhance product quality and regulatory compliance, accelerate regulatory approval and market access, optimize supply chains and mitigate the shortage of skilled workers along the production value chain. The ongoing evolution of AI is expected to create transformative opportunities for early adopters in the in vitro diagnostic sector, where those overcoming existing barriers may gain substantial competitive advantages and contribute meaningfully to the enhancement of local production ecosystems. Future integration of AI into local in vitro diagnostic manufacturing in low- and middle-income countries could be realized through a multifaceted, multistakeholder approach, including strategy and policy support, targeted investments, effective technology transfer, tailored capacity-building, predictable market demand, international collaborations, and blended funding mechanisms. By pursuing these strategic approaches, low- and middle-income countries would not only accelerate the adoption of AI in in vitro diagnostic manufacturing but also help to close the technology access gap during this transformative era, fostering more equitable access to AI-driven advancements in diagnostics.

## References

[1] De Leon RP, Tejero LMS. Medical device development from ideation to regulation and technology transfer in low- and middle-income countries. Acta Med Philipp. 2023 Jun 28;57(6):70–6. 39483686 10.47895/amp.vi0.5134 PMC11522637

[2] Khinvasara T, Ness S, Shankar A. Leveraging AI for enhanced quality assurance in medical device manufacturing. Asian J Res Comput Sci. 2024;17(6):13–35. 10.9734/ajrcos/2024/v17i6454

[3] Data and AI are helping to get medicines to patients faster [internet]. New York: Pfizer; 2023. https://www.pfizer.com/sites/default/files/investors/financial_reports/annual_reports/2022/story/data-and-ai-are-helping-to-get-medicines-to-patients-faster [cited 2025 Aug 21].

[4] Ethics and governance of artificial intelligence for health: guidance on large multi-modal models. Geneva: World Health Organization; 2024. Available from: https://iris.who.int/handle/10665/375579 [cited 2025 Jan 26].

[5] Awaisi KS, Hussain S, Ahmed M, Khan AA, Ahmed G. Leveraging IoT and fog computing in healthcare systems. IEEE Internet Things Mag. 2020 Jun;3(2):52–6. 10.1109/IOTM.0001.1900096

[6] Mateev M. Predictive analytics based on digital twins, generative AI, and ChatGPT. In: Callaos N, Gaile-Sarkane E, Hashimoto S, Lace N, Sánchez B, Savoie M, editors. Proceedings of the 27th World Multi-Conference on Systemics, Cybernetics and Informatics: WMSCI 2023; 2023 Sep 12–15; virtual conference. Winter Garden: International Institute of Informatics and Cybernetics; 2023. pp. 168–74. 10.54808/WMSCI2023.01.168

[7] Khlie K, Benmamoun Z, Jebbor I, Serrou D. Generative AI for enhanced operations and supply chain management. Journal of Infrastructure, Policy and Development. 2024;8(10):6637. 10.24294/jipd.v8i10.6637

[8] Reniewicz J, Suryaprakash V, Kowalczyk J, Blacha A, Kostello G, Tan H, et al. Artificial intelligence / machine-learning tool for post-market surveillance of in vitro diagnostic assays. N Biotechnol. 2024 Mar 25;79:82–90. 10.1016/j.nbt.2023.11.005 38040287

[9] Regulatory considerations on artificial intelligence for health. Geneva: World Health Organization; 2023. Available from: https://iris.who.int/handle/10665/373421 [cited 2025 Jan 26].

[10] Ethics and governance of artificial intelligence for health: WHO guidance. Geneva: World Health Organization; 2021. Available from: https://iris.who.int/handle/10665/341996 [cited 2025 Jan 26].

[11] Addressing future cybersecurity threats in digital health: report of the technical consultation, Geneva, Switzerland, 12-14 December 2023. Geneva: World Health Organization; 2021. Available from: https://iris.who.int/handle/10665/379097 [cited 2025 Jan 26].

[12] Holzinger A, Zatloukal K, Müller H. Is human oversight to AI systems still possible? N Biotechnol. 2025 Mar 25;85:59–62. 10.1016/j.nbt.2024.12.003 39675423

[13] Resolution WHA74.6. Strengthening local production of medicines and other health technologies to improve access. In: Seventy-fourth World Health Assembly, Geneva, 24 May–1 June 2021. Geneva: World Health Organization; 2021. Available from: https://apps.who.int/gb/ebwha/pdf_files/WHA74/A74_R6-en.pdf [cited 2025 Jan 26].

